# Periodic liquid crystalline waveguiding microstructures

**DOI:** 10.1038/s41598-023-41255-6

**Published:** 2023-08-25

**Authors:** Sławomir Ertman, Kamil Orzechowski, Katarzyna Rutkowska, Oliwia Kołodyńska, Julia Różycka, Adam Ignaciuk, Natalia Wasilewska, Tomasz Osuch, Tomasz R. Woliński

**Affiliations:** 1 Faculty of Physics, Warsaw University of Technology, Koszykowa 75, 00-662 Warsaw, Poland; 2 Faculty of Electronics and Information Technology, Institute of Electronic Systems, Warsaw University of Technology, Nowowiejska 15/19, 00-665 Warsaw, Poland

**Keywords:** Fibre optics and optical communications, Liquid crystals

## Abstract

Different methods allowing for creating optical waveguides with liquid–crystal (LC) cores, in which molecules form periodic patterns with precisely controlled periods, are reported. The first one is based on reversible photoalignment with high-resolution selective illumination and allows to control the period of LC molecules inside silica microcapillaries. The second method employs microstructures formed in PDMS, allowing to obtain both: LC-core waveguides and a set of specially designed periodic microelectrodes used for the periodic reorientation of molecules. Using both methods, we successfully controlled the period of the patterned alignment in the range from about 500 µm and scaled it down to as small as 20 µm. We performed experimental studies on waveguiding phenomenon in such structures, in view to obtain transmission spectra typical to optical fiber gratings. Since the results achieved in experimental conditions differed from those expected, the additional numerical simulations were performed to explain the observed effects. Finally, we obtained the waveguiding in a blue phase LC, characterized by naturally created three-dimensional periodicity with periods smaller than one micrometer. In such a structure, we were able to observe first-order bandgap, and moreover, we were able to tune it thermally in nearly the whole visible spectral range.

Photonics-based technologies hold an enormous potential to revolutionize the twenty-first century as electronics did in the twentieth century. They can offer an important level of miniaturization and integration to achieve enhanced functionalities simultaneously with efficient power consumption.

Liquid crystal (LC) planar waveguiding structures have been investigated over the last few decades^1,2^, forming a novel platform that addresses the need for an integrated configuration suitable for tunable devices. Extremely high electro-optic responses and thermo-optic effects in LCs, combined with their high birefringence and large dielectric anisotropy, result in an extraordinary potential in application to waveguiding structures. In confined structures, such as, e.g., microcapillary tubes infiltrated with LCs, waveguiding effects in both circular- and elliptical-core LC fibers have been reported^3^. The elliptical-core (4 × 18 µm) LC cylindrical waveguide was found to be an unusual example of a multimode single-polarization optical fiber^4^.

Another type of LC waveguiding structures based on photonics crystals fibers (PCFs) are photonic liquid crystal fibers (PLCFs)^5–8^ also known as LC-infiltrated PCFs. PLCFs are advanced specialty fibers that benefit from a combination of “passive” PCF host microstructures infiltrated with “active” LC guest materials and are responsible for a variety of their unique properties. PLCFs create a novel class of optical waveguides that utilize exceptional guiding properties of the solid-core PCFs and attractive tunable properties of the LC photonic microstructure in the fiber cladding. LC-infiltrated PCFs introduce new levels of tunability to PCFs and boost their performance due to a diversity of novel propagation, spectral, thermo-optic, electro-optic, and polarization properties. Apart from their high sensitivity to temperature and to electrical/magnetic/optical fields, the use of different liquid crystal molecular orientation “scenarios” within the microholes can determine either index guiding or photonic band-gap propagation mechanisms, as well as reversible switching between them.

Periodic waveguiding structures have played a vital role in the evolution of photonics. In addition to transversely periodic structures (e.g., photonic crystals and photonic crystal fibers), waveguiding structures in which the refractive index varies periodically along propagation direction are also important. Their impact extends over a wide range of functionalities of photonic devices, including grating coupling, (Bragg) reflection, polarization conversion, deflection, second harmonic generation, frequency modulation, and more. The wide variety of the devices results from the different ways in which the waveguide geometries, periodicity (described by duty cycle and refractive index modulation/difference), coupling effects, and guided modes may be chosen.

Periodic photonic microstructures have been investigated in recent years for waveguiding applications (e.g., Bock, PJ et al*.*^9^) because of their potential uses as modulators, filters, or photonic integrated elements. At the same time, two main types of fiber gratings are distinguished in fiber optics: long-period fiber gratings (LPFGs) and fiber Bragg gratings (FBGs). Although they both involve the creation of periodic variations in the fiber structure, they have distinct differences in their structures and operating principles. The LPFGs performance is mainly based on the coupling between the core and cladding modes of the fiber, and its period is typically in a range from few hundreds micrometers to even few millimeters^10^. The FBGs operate by taking the adventage of the Bragg reflection phenomenon, thus, their periods are usually in a range of the wavelength, and transmission spectra have much narrower bandwidths^11^. Both mentioned types of gratings find many interesting applications, including sensing, mode convertion, and wavelength-specific applications (lasers, filters, add-drop wavelength division multiplexing etc)^11,12^. Potential applications of such gratings could be significantly expanded if the gratings characteristics are effectively controlled, allowing for repeatable and fully predictible tuning of their transmission spectra. One of the potential strategies to obtain such solution, is to combine the fiber grating with thermo-optic polymer^13^ (or fluid), but it has to be kept in mind that thermal tuning is not the best solution for the practical devices^14^.

It seems that a more attractive path is to marge fiber gratings with liquid crystals. The first attempt to combine LPFG with LC has been reported in Ref.^15^, where LC-core fiber was tuned with periodic electrodes (with a period of 483 µm), allowing for a band rejection with the bandwidth of about 15 nm. Such an achievement was accomplished with a relatively small tuning range (up to 6 dB), requiring the steering signal of 250 V. Even if theoretical simulations showed that dynamics of such structures could reach 20 dB^16^, it has not been verified experimentally so far. Also, results of PCFs filled with LCs with electrodes’ period of 800 µm^17^ as well electrically and mechanically induced LPFGs^18^ (also with period of 800 µm) were reported. A different approach was present in Refs.^19,20^, where “standard” LPFGs were surrounded with thin layer of LC, allowing for both thermal and electric tuning of the position of the transmission spectra dip.

Another interesting approach was presented in Ref.^21^, where a solid-core PCF was filled with UV-curable adhesive and then selectively exposed to UV light by using three different masks with periods of 600, 500 and 400 µm. Since the refractive index of the used adhesive (NOA75) is higher than the refractive index of the silica glass, the obtained transmission spectra combined two different PBGs characteristics with spectra typical for LPFG. Such a combination of transmission characteristics resulted in narrower bandgaps with attenuation increased by about 7 dB. The signal recorded when using two masks (with periods of 600 and 400 µm, respectively) contained notches (about 5 dB depth) typical for LPFG. A similar combination of PBG and LPFG was presented in Ref.^22^, where solid-core PCF was filled with E7 LC mixed with 25% of 4MAB (4-methoxyazobenzene). Such a mixture could be reversibly tuned with light (due to the isomerization of 4MAB), so it was possible to observe a notch with depth of about 15 dB when using the mask with a period of 700 µm. However, since the authors have not used masks with periods other than 700 µm, it is unclear if the notch in the transmission spectra results from the formation of LPFG or it is an effect of the superposition of two PBGs (similarly like in Ref.^23^). Another worth noting type of photo-induced fiber grating is reported in Ref.^24^, where so-called polymer–liquid crystal polymer slices (POLICRYPS) were holographically recorded in selectively filled HB-PCF with a period as low as 2 µm. Even if authors did not manage to record the spectra typical for Bragg grating, it was the very first demonstration of such a small periodicity inside PCFs.

Considering the important role of tunable periodic waveguiding structures in modern photonic systems, this paper describes three new types of microstructures with liquid crystalline materials both in micro-capillaries and in planar structures. In particular, three scenarios of periodic LC waveguiding microstructures creation are presented that are based on (1) periodically aligned nematic LC-core fiber microstructure confined in a silica micro-capillary, (2) LCs with polydimethylsiloxane (PDMS) and (3) Blue Phase Liquid Crystal (BPLC). Periodicity in the investigated LC waveguiding microstructures is induced respectively by: (1) reversible photo-induced molecular alignment, (2) specific electrically driven microchannels infiltrated with nematic LC, or (3) inherent self-assembling LC cubic structures.

## Methods

### Experimental setup for the reversible generation of periodic alignment of liquid crystal molecules in micro-capillaries

**Figure 1:**
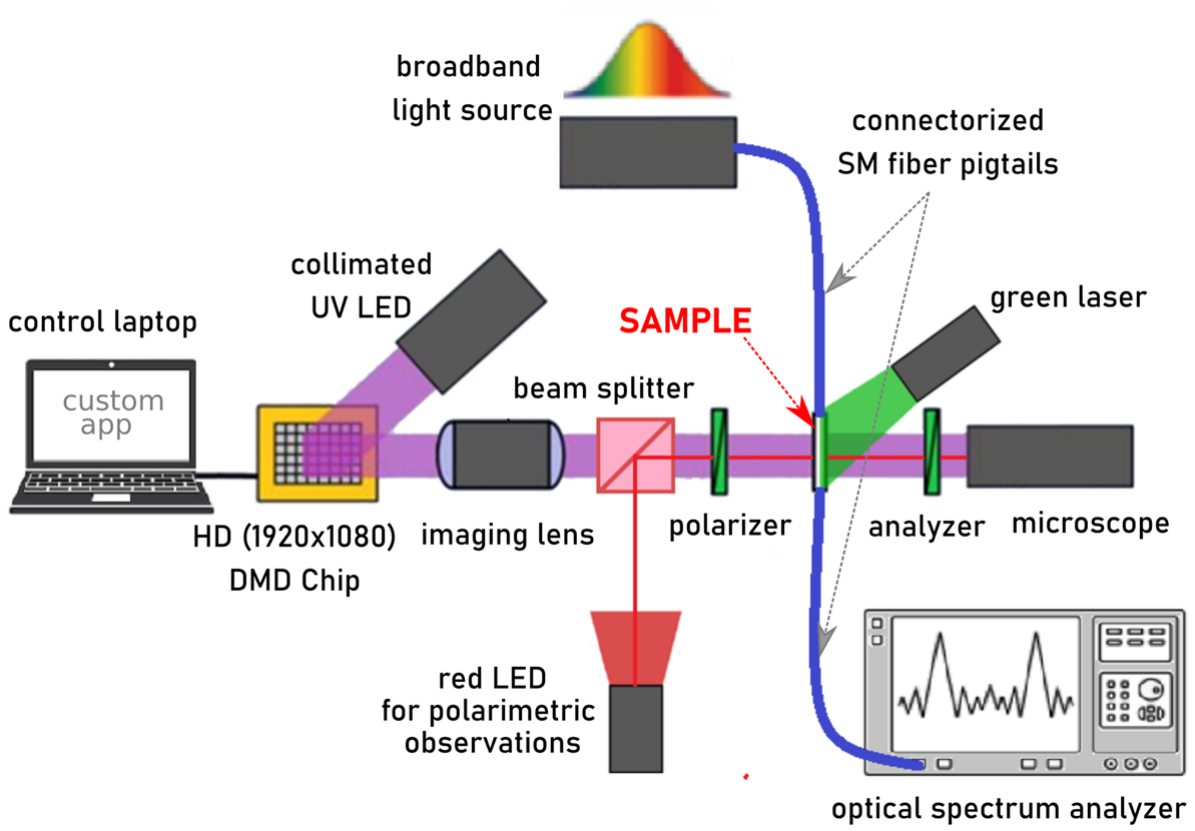
Experimental setup for the reversible generation of periodic alignment of liquid crystal molecules in micro-capillaries.

In order to generate periodic alignment in silica microcapillaries, we used a well-known photoalignment technique^25^. It has already been demonstrated that it is possible to generate periodic alignment by using multi-step irradiation with the use of amplitude masks, allowing for periods in the order of hundreds of micrometers^26,27^. In this work, we used two commonly-known photo-aligning azo-dyes: SD1 and BY (brilliant yellow)^28,29^. We noticed that obtained results were remarkably similar to both materials. The sample preparation procedure was also almost the same for both materials: we used a 5% solution of azo dye in DMF to fill the capillaries. Next, high-pressure (~ 2 atm) air was used to pull out most of the solution from the capillary. Finally, capillaries were baked for 30 min at 120 °C. After this step, a thin layer of azo dye was left on the inner walls of the microcapillary, which was ready to be filled with nematic liquid crystal (5CB). Next, a short section (few millimeters long) of the sample was connected with two connectorized standard single-mode optical fibers (SM128). The prepared sample was carefully placed in a specially designed setup (Fig. 1).

To obtain periodic patterns of light needed for photoalignment, we used Digital Micromirror Device (DMD) chip DLP4710 from National Instruments, allowing for full HD resolution (1920 × 1080 pixels). The DMD chip was illuminated with a highly collimated LED with a central wavelength of 405 nm. To obtain photo-aligning effect, the beam of light has to be polarized, so we placed a polarizer directly before the sample. We also used an optical imaging setup to obtain an image of the DMD chip in the plane where the sample was placed. The size of the pixel of the DMD chip was 5.4 µm, but our imaging setup allowed us to scale it down to 2.5 µm. The quality of imaging was continuously monitored with a digital microscope Keyence VHX-5000. In such a setup generation of periodic alignment of the LC molecules was quite simple and well-repeatable—different periods were easily obtained just by changing the pattern of illuminating image. Recording of periodic orientation consisted of two steps: first polarizer axis was set to 90 degrees (with respect to the axis of the sample), and the sample was illuminated with a generated periodic pattern for about 2–5 min. Next polarizer was rotated by 45 or 90 degrees, and the sample was illuminated for the second time with the periodic pattern (reversed to the one used for the first illumination). In order to erase periodic orientation within the sample, we used two different techniques: one based on long illumination with unpolarized 405 nm light (or uniformly polarized light to erase the sample and induce uniform alignment), and the other based on illumination with green laser (532 nm, 100 mW, focused to 10 mm line), which was used to speed up cis–trans isomerization of the azo dye. In some cases, we used two methods simultaneously. Since the sample was sensitive for the light from UV to green spectral range, we used polarizing microscopy imaging (Keyence VHX-5000) with red light illumination for analyzing the orientation of the LC molecules in micro-capillaries. It is worth mentioning that each subsequent erasing and recording of new alignment required slightly more time, and thus the orientation quality was decreasing—i.e., the borders between two different orientations were slightly more blurred and less stable. Based on our previous studies^26,30^, we can confirm that the stability of the alignment of LCs obtained with photoalignment technique is excellent and the samples (stored in UV-free conditions) “remember” recorded periodic alignment of molecules even after the period as long as 24 months. It is also worth mentioning that photoalignment is probably the most suitable technique to use when dealing with microcapillaries. The use of other techniques (e.g., the high-resolution plasma of focused ion beams) is strongly limited or even impossible (due to the access to the oriented surface is physically blocked by the walls of the capillary).

### Numerical design and practical preparation of periodic waveguides in LC:PDMS

**Figure 2:**
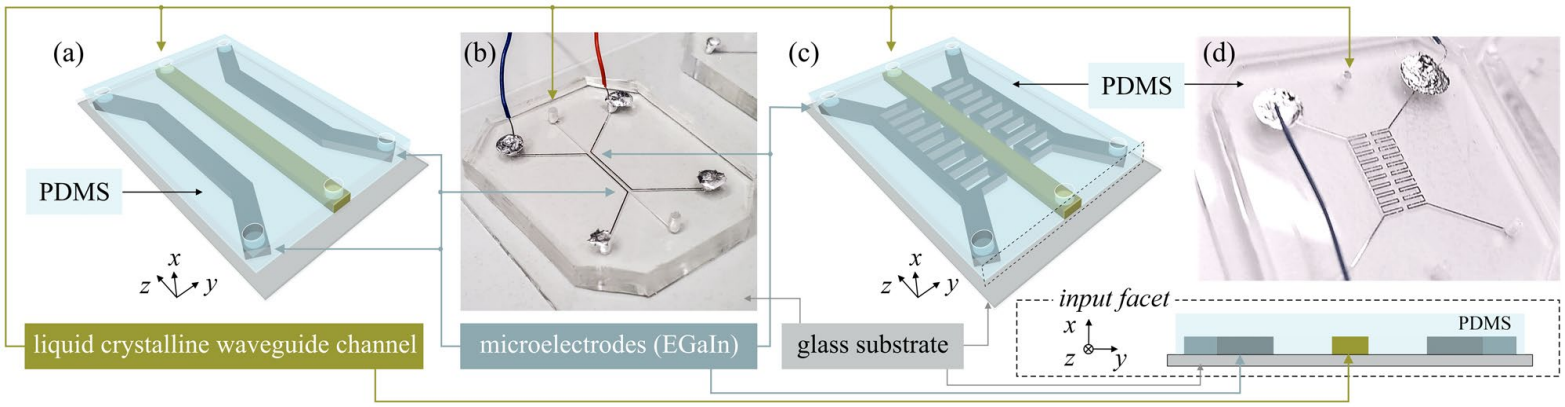
Schemes (**a,c**) and photos (**b,d**) of the electrically tunable LC: PDMS waveguiding structures with microelectrodes made of EGaIn. Inset in the bottom-right corner shows the input facet of the structure.

We created LC:PDMS structures (see Fig. 2) using the typical cast-and-molding technique with the mold and waveguiding device fabrication processes described in our previous papers^31–33^, respectively. It must be underlined that all the samples presented in this work were obtained from high-quality molds fabricated using the photolithography process with UV illumination through the chrome mask^33^. Specifically, SU-8 25 and SU-8 2010 negative photoresists (provided by Kayaku Advanced Materials Inc.) have been applied, providing a high-aspect ratio with nearly vertical sidewalls of the rib to be embossed in PDMS^34,35^. Following the fabrication procedures described in Refs.^31–33^, it was possible to obtain the channels with a height of about 30 µm (for the structures shown in Fig. 9a–c) and 12 µm (for the structure shown in Fig. 9d), respectively. A detailed description enriched with the photos taken with Hitachi SU-8230 SEM to verify the quality of the structures in the PDMS is presented in our previous publication^32^. The roughness of the sidewalls of the PDMS channels fabricated using the SU-8 mold was negligible (< 400 nm). Moreover, for the mold of the height of 12 µm it was possible to reduce the width of the channels to the single micrometers (limited mainly by the possible mechanical damage when detaching the PDMS chip from the mold). It has to be noted that the structures shown in this paper are not of the smallest in size that have been achieved, while the primary goal of this communication was to demonstrate the principle of operation in the structure with the high contrast ratio between the regions of different molecular orientation when the voltage is applied. Referring to other illumination techniques presented in this paper, it is worth mentioning that the DMD-based maskless lithography in SU8 may also be considered as the potential option for the mold fabrication^36^, but the waveguide channels' transverse sizes cannot be reduced to the values obtained in this work (being in the range of several tens of micrometers^37^).

Theoretical and experimental results regarding LC:PDMS structures presented here have been obtained for typical E7 LC and Gallium-Indium eutectic, EGaIn (both commercially available from Merck) used to fill the appropriate microchannels. We performed numerical simulations using custom scripts based on the ADSOR scheme for molecular reorientation (using equations and methodology described in our previous papers^38,39^) and the COMSOL Multiphysics software to calculate the electric field distribution.

### Formation of BPLC microcavity in microstructured fiber

**Figure 3:**
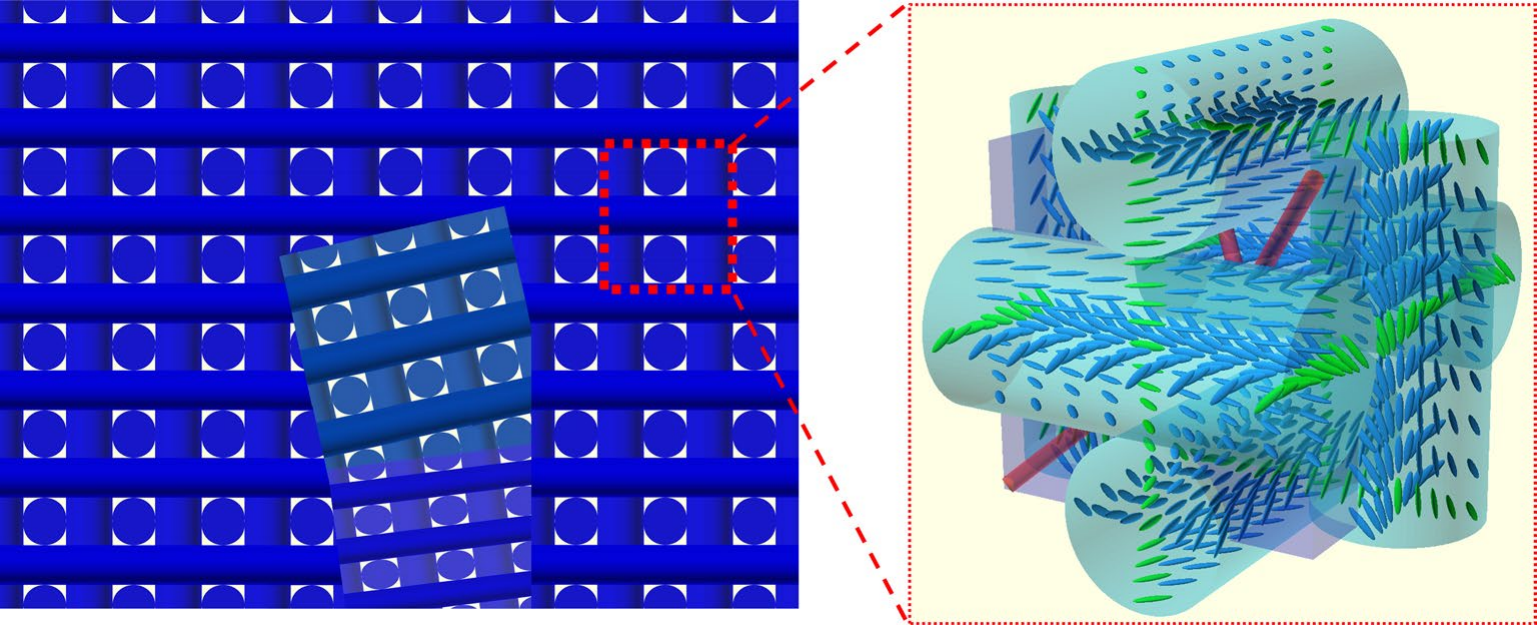
Schematic formation of a polydomain BPLC sample (left) and molecular ordering in a cubic unit cell of BPII (right). Green molecules mean connected helices in neighboring double-twist cylinders, and the red rods represent an array of LC disclinations in a unit cell.

In the experiment, we used BPLC formed by doping nematics with a strongly twisting chiral dopant. The major compositions of the LC mixture are nematics (82.6 wt. %) that are photochemically stable fluorinated oligophenyls with fluorinated cyclohexyl- and bicyclohexylbiphenyls. BPs were induced in the investigated LC mixture by adding two chiral dopants: biphenyl-4,4-dicarboxylic acid bis(1-methyl heptyl) ester (8.7 wt. %) and [1,1;4,1] terphenyl-4,4-dicarboxylic acid bis(1-methyl heptyl) ester (8.7 wt. %). All components were synthesized at the Institute of Chemistry of the Military University of Technology^37,38^. To prepare the BPLC microcavity in the fiber sample, a microcapillary with an inner diameter of 128 μm was carefully filled with the BPLC mixture. Then, a multimode fiber with an outer diameter of 125 μm was introduced to the microcapillary and pushed until it came into contact with the BPLC filling. It is worth noting that without applying any alignment layers or external electric fields, BPLC tends to align only for a small thickness. For such a thick layer as tens of micrometers, BPLC may exhibit nonuniform structures revealing a polydomain sample, what is schematically presented for BPII in Fig. 3. To achieve stability of the entire sample and to prevent leakage of BPLC, the optical adhesive (NOA 65, Norland) was coupled into free space between microcapillary and multimode fiber. Then it was cured by a high-power UV light (~ 200 mW/cm^2^, Dymax UV Lamp) for 30 s. This process was done under a polarized microscope and was repeated for the second multimode fiber to cover the BPLC microcavity on both sides. Measurements of transmission properties of the investigated microstructured fiber with the BPLC microcavity were performed with the use of Ocean Optics FLAME-S VIS–NIR spectrometer and Schott KL 1600 LED source with mounted the objective (20 × , NA = 0.4) for coupling the light into the fiber sample.

### Numerical simulations of transmission spectra of LC-core waveguides with periodic molecular alignment

Analysis of the influence of the “non-ideality” of a one-dimensional photonic structure produced by selective UV irradiation on its spectral characteristics. By “non-ideality” we understand a non-ideal molecular alignment and fluctuations of molecules that could result in random changes of the refractive index, but also the length of the ordered sections or even a period.

A custom-made software for calculating spectral characteristics of one-dimensional photonic structures with the possibility of implementing scatters of structure parameters was developed. The software considers the possibility of introducing variations of such parameters as: a period of the grating, filling factor of the grating (ratio of widths of the regions with two different alignments of molecules), and thus with different refractive indices.

We assume that the studied structure consists of alternating regions with refractive indices n_1_ and n_2_ and corresponding widths of w_1_ and w_2_. Thus, the period of the structure is P = w_1_ + w_2_. Further assumptions for layers of the periodic structure are isotropy, homogeneity (constant refractive index within a particular layer), and loss lessness (i.e., only the real part of the refractive index was considered). It was also assumed that the incident optical wave propagates in the direction perpendicular to the boundary of the consecutive sections of the periodic structures, and therefore, considering the isotropy of the layers, the calculated transmission spectra obtained for the TE- and TM-polarizations of the incident wave are identical.

Since the main aim of the simulations was not an accurate model of spectral characteristics of the examined samples but to demonstrate how the instability of structure parameters affects changes in the transmission/reflection spectrum, the use of the simplified structure model (described above) allows to obtain reliable results.

## Results

### Spectral properties of microcapillaries filled with periodically aligned liquid crystals

**Figure 4:**
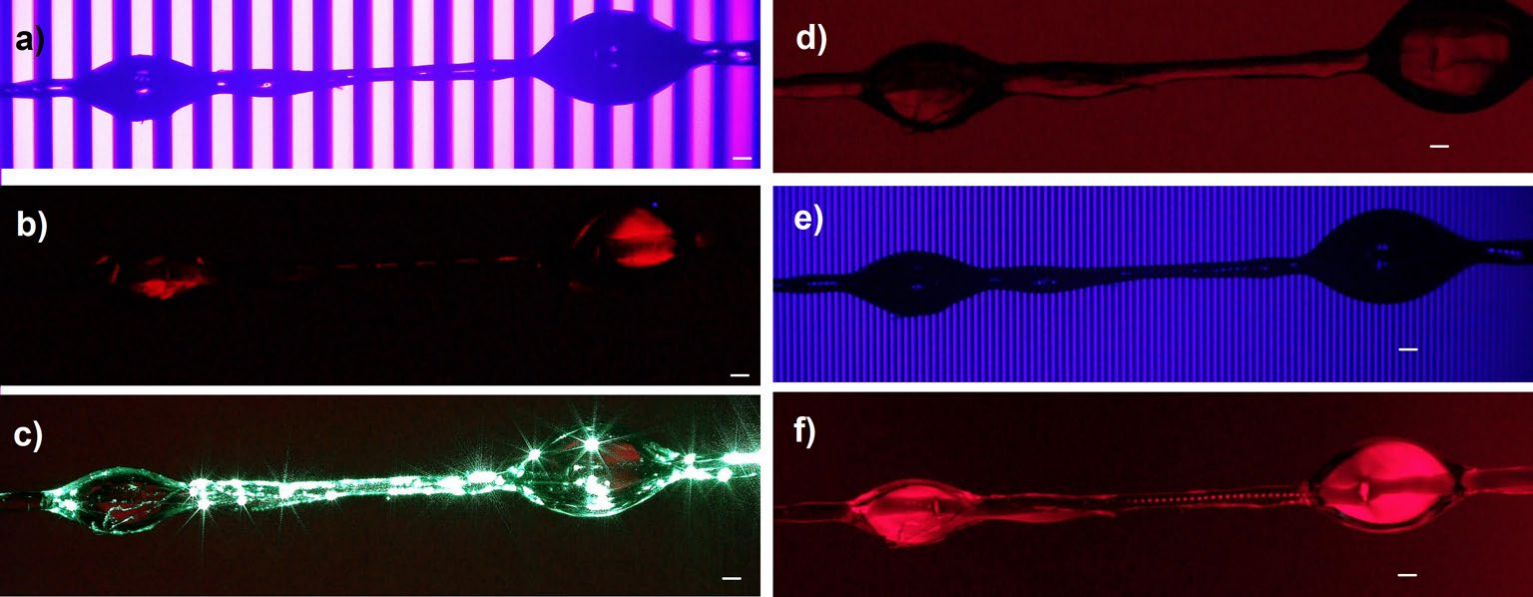
An exemplary sequence of recording and erasing of various periodic alignments of liquid crystal molecules: periodic pattern of linearly polarized UV light is used to generate alignment (**a**), then unpolarized UV light*,* optionally assisted with green laser (532 nm) can be used to erase alignment (**c**), and another periodic pattern could be used for illumination (**e**). After each step of UV illumination, the resulting alignment of the LC molecules is analyzed with polarizing microscopy (**b**,**d**,**f**) illuminated with red light (which is “neutral” to the sample). The white scale bars presented in each photo correspond to 100 μm.

We prepared a number of the samples based on silica microcapillaries filled with the 5CB liquid crystal. We used microcapillaries with four different inner diameters (6, 9, 12, and 20 µm) and external diameter of 125 µm. By using reversible photoalignment technique (described in the Methods section), we were able to generate periodical changes of the alignment of the LC molecules (recording of the grating). Due to the reversibility of the photoalignment the grating could be erased and then recorded again, i.e., with a new period. Our setup allowed us to change the period from about 10 to 800 µm (larger periods were possible but not tested). Figure 4 shows an exemplary sequence of recording and erasing of the grating. First, the sample is illuminated with the first periodic pattern of polarized UV light (Fig. 4a), then the obtained alignment is examined between two crossed polarizers (Keyence VHX-5000 digital microscope) by using “neutral” red light with the wavelength higher than 640 nm (Fig. 4b). If the alignment has confirmed the periodicity, transmission spectra have been measured with Yokogawa AQ 6370C in the range from 1200 to 1600 nm (using two Thorlabs SLEDs: S5FC1021P + S5FC1005P). After characterization of the grating, it can be erased by using eighter the unpolarized uniform UV light, or the UV light assisted with a green laser (propagating at the wavelength of 532 nm, 100mW), see Fig. 4c, with an erasing process again controlled by using the "neutral" red light (Fig. 4d). Finally, the sample was irradiated with the second periodic UV pattern characterized by a different period (Fig. 4e) and the resulted alignment of the LC molecules was again examined with red light illumination (Fig. 4f).

**Figure 5:**
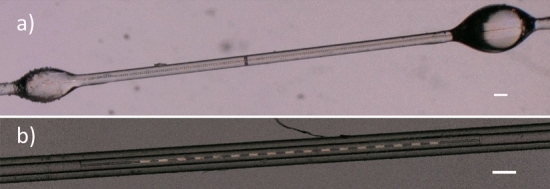
Exemplary pictures of optimized samples with the stable and well-defined periodic alignment of the LC molecules—(**a**) the sample created using direct gluing of LC-filled capillary to the SM-fibers and (**b**) the sample created by placing LC-filled capillary and both SM-fibers in a larger capillary, with an inner diameter of about 126 µm. The white scale bars in both pannels correspond to 100 μm.

We created and analyzed a substantial number of similar samples to optimize the entire process of LC molecules’ periodic alignment generation. We also tried to optimize the coupling of light between LC-filled capillary and SM fibers—i.e., by minimizing the amount of air at the ends of the sample and by a careful and precise alignment. Examples of such optimized samples are included in Fig. 5. In our experiments, we used two different techniques for connecting SM fibers with the LC-filled capillary by using precise gluing with UV-curable glue (Fig. 5a), and an additional external capillary with the inner diameter of about 126 µm (Fig. 5b). The second method is less accurate. However, we decided to use it to eliminate the risk of unwanted degradation of the sample during photopolymerization of the UV-curable glue (moreover, later we managed to optimize a gluing method, and only small spot of UV-curing light was used to illuminate small droplet of the glue).

**Figure 6:**
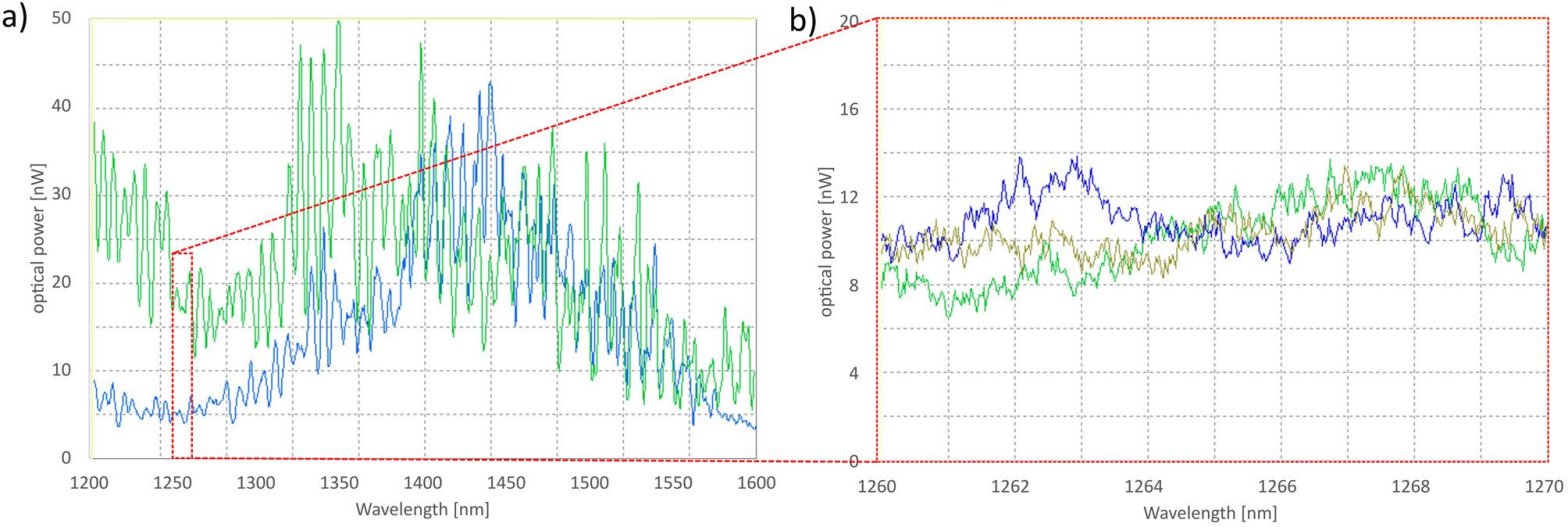
Exemplary (and typical for most samples) transmission spectra recorded for optimized samples with periodic alignment of liquid crystal molecules. All spectra were recorded for the same sample but at different moments. It could be noticed that spectra are not only noisy but also highly unstable in time. Both (**a**) and (**b**) panel present results recored for the same sample, however (**b**) is “zoomed” to range from 1260 to 1270 nm, to show that “noise” is very random.

In our research, we measured transmission spectra for each created sample. We were expecting that it would be possible to observe dips in the transmission spectrum (similar to those observed for typical fiber gratings, i.e., Bragg or long-period fiber gratings). Unfortunately, even for very carefully optimized samples we could not observe expected spectra. The first impression after looking at exemplary samples presented in Fig. 5 is that it should result in “grating-like” spectra. However, we were not able to observe any characteristic transmission dips that could be undoubtfully identified as resulted from the grating. In most measurements, we observed quite “noisy” spectra, which were moreover highly unstable in time (see Fig. 6), but without any noticeable transmission dips – we expected not only to observe such dips but also to change its position by varying the grating period.

**Figure 7:**
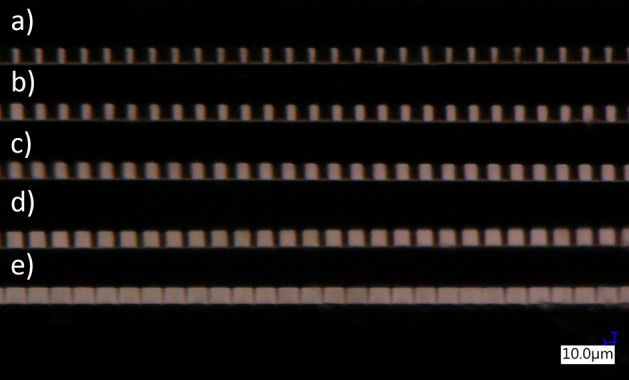
Examples of the LC-filled capillary with periodic alignments and variable filing factor – the period for each sample is about 23 μm and corresponds to 10 pixels of DMD chip. Changes of filling factor were obtained by changeling the number of pixels used for illumination in each step, equal to about: (**a**) 15% (~ 3.5 μm); (**b**) 30% (~ 7 μm); (**c**) 50% (~ 11.5 μm); (**d**) 70% (~ 16 μm) and (**e**) 90% (~ 21 μm), respectively.

It is worth mentioning that we were able not only to change the period of the LC grating, but we were also able to control the filling factor of the grating (by adjusting the widths of the sections of different molecular alignment within the same period – see example in Fig. 7). However, the results were similar to the mentioned above, and we still were not able to obtain “grating-type” spectra. Although analysis of the periodic alignment of LC molecules with a polarizing microscope was highly optimistic, it seems that there is a need to make more extensive efforts to obtain stable and tunable “grating-type” spectra. Possible explanations for the difficulties encountered are described in the “Discussion” Section.

### Tunable periodic orientation of LC molecules in PDMS:LC waveguides

In our work, we have also designed, fabricated, and assessed experimentally planar waveguides made of LC-core embedded in PDMS material. The periodic microelectrodes were used to obtain local reorientation of the LC molecules and, thus, the periodic orientation of LC molecules (and periodic refractive index modulation) within the core.

The combination of LC materials with PDMS is of particular scientific attention when it comes to the practical realization of the optofluidic devices^40,41^, including waveguiding structures^42–49^, enabling potential applications as, e.g., electro-optically-controlled and reconfigurable interconnectors^50^, switches^45^, multimode interference couplers^51^, and wavelength multi/demultiplexers^45,50,51^. A significant advantage of such a concept stem from the low-cost and simple elastomer processing and the high tunability of optical properties, which may be realized electrically. Designs of any shape, including complex patterns with a resolution down to single micrometers, may be easily achieved. Moreover, the spontaneous vertical alignment (VA)^33^ of LC molecules on the PDMS surface simplifies the fabrication procedures, making additional orienting layers needless. The primary concern when fabricating electrically driven structures, especially with sophisticated geometry, is creating a suitable and efficient electrodes' system. A significant challenge is depositing or introducing the conductive elements on or into the PDMS slab. Pattering metallic structures, commonly used in microelectronics, is greatly hindered due to the low adhesion and low surface energy of PDMS. Besides different attempts, including thin metallic layers deposition on PDMS surface^52,53^, coating with conductive polymer^54^, or adding metallic nanoparticles into PDMS volume^55^, the recent developments have been focused on electrically-tunable LC:PDMS waveguiding structures formed by the microchannels (embedded in PDMS slab bounded to the glass substrate) and filled with liquid crystalline and fluid conductive material, respectively. Such a configuration allows for complex electrodes to be realized and placed in the close vicinity of the waveguiding channel. Considering the main topic of this work, it is worth noting that periodic waveguiding structures may be relatively easily achieved with use of electrodes of special design and geometry^31^.Specifically, a concept of fluid electrodes, allowing for periodic changes of refractive index along the liquid crystalline waveguide channel^31^, brought the idea of the possible application of LC:PDMS chips as the photonic Bragg-type structures.

**Figure 8:**
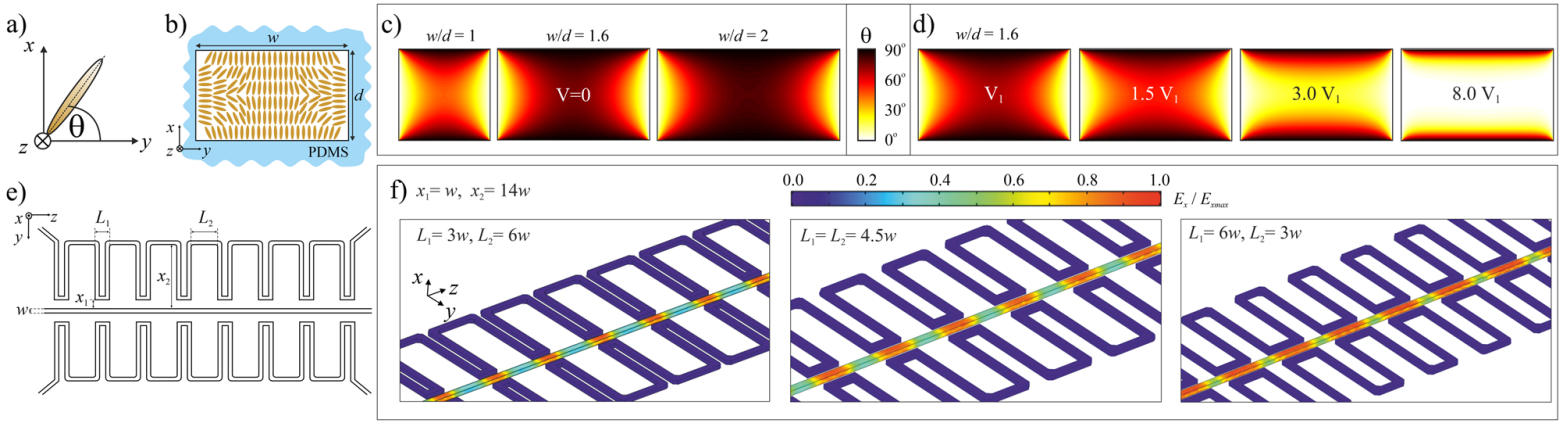
Definition of the orientation angle (**a**). Schematic representation (**b**) and results of numerical simulations (**c**) showing the LC molecular alignment within the cross-section of the waveguide channels (with different ratio between their widths, *w* and heights, (**d**) without voltage. Electrically driven changes in molecular orientation and thus in the spatial refractive index distribution retrieved numerically when assuming the flat electrodes placed along vertical walls of the liquid crystalline channel with the size ratio of *w*/*d* = 1.6 (**d**). Definition of the geometrical parameters of the periodic electrodes (**e**) and normalized electric field distribution (for *x*-component) determined within liquid crystalline waveguide channel for periodic electrodes with different ratios for the lengths of the close and far sections (**f**).

Numerical simulations have been performed to design waveguiding structures with periodic refractive index modulation along the propagation direction. They include calculations regarding molecular arrangement within waveguide channel cross-section depending on the electric voltage applied (see Fig. 8a–d) and electric field distribution along the waveguide channel when the periodic electrode of specific shape (i.e., with a particular length of subsequent segments and their distance from the waveguide channel, see Fig. 8e,f).

It has been demonstrated that the vertical anchoring of the rod-like NLC molecules on the PDMS surface is spontaneously achieved in stationary conditions^42,56^. Such molecular arrangement is related to the high hydrophobicity and low surface energy of the elastomer, and, in the case of the structures analyzed, it is consistent with a homeotropic orientation on each of the channel walls after the NLC flow termination^57^. The high morphological stability characterizes the nematic textures obtained in this way. The resultant alignment of NLC molecules within PDMS-embedded microchannels strongly depends on the channels' aspect ratio^58^, the quality of the molds used in the fabrication process^33^, and the method of connecting the PDMS chip to a substrate^59^. Based on previous studies^32,33,60,61^, it has been concluded that the best results in terms of predictability, reproducibility, robustness, and stability of LC molecular alignment in LC:PDMS structures are obtained for the molds obtained in the photolithography process^33^ and with the assistance of the plasma treatment, reducing the PDMS surface roughness^60,61^. In fact, such procedures have been applied when fabricating the samples shown in this paper. The vertical alignment at the PDMS surfaces could be affected thermally or by the introduction of the flow. However, it could be retrieved after cooling the sample to the temperature specific for the nematic phase and by stopping the circulation of the liquid crystalline material within the channels.

**Figure 9:**
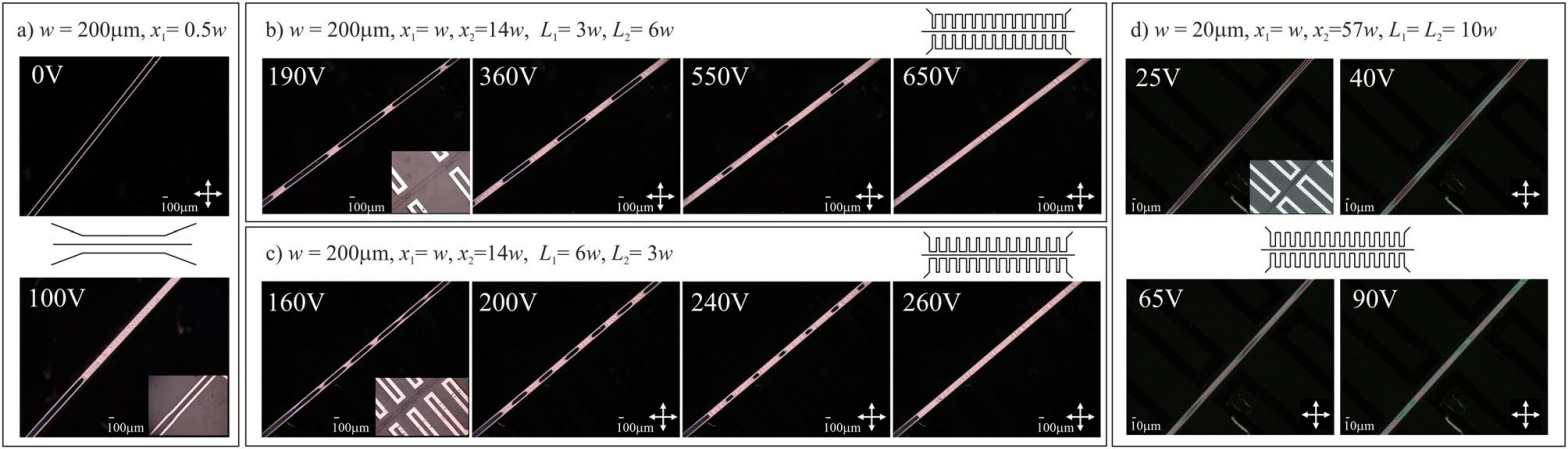
Experimental results in the form of photos taken with the use of the polarizing microscope (in the transmission mode with crossed polarizers) showing the change in LC molecular alignment obtained by applying the voltage to the pair of conventional (**a**) and periodic (**b–d**) microchannel electrodes infiltrated with GaIn eutectic. Insets in panels show the structure geometry. The geometrical parameters of each structure are given above the photos. Schematic drawings presenting the structures are also shown for clarity.

**Figure 10:**
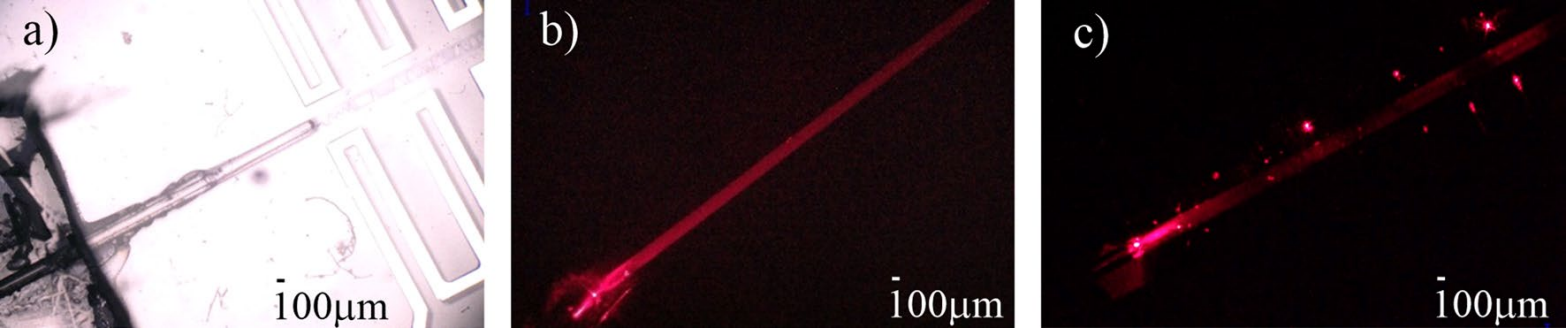
Photos taken in experimental conditions showing the optical fiber introduced into the liquid crystalline channel (**a**) and light propagation for no voltage (**b**) and reorienting voltage (of about 200 V) (**c**) are applied. In the presented case, the width of the channel was of about 200 µm allowing thus for inserting the polymer optical fiber into the liquid crystalline waveguide channel.

Experimental results showing the electric tunability of LC:PDMS waveguiding structures are shown in Fig. 9. Specifically, the structures with homogeneous and periodical electrodes are presented. Please note that the bright color within the central channel corresponds to the situation in which most LC molecules are aligned along the electric field direction, which is perpendicular to the waveguide axis. It is important to note that the length of the reoriented segment changes with applied voltage up to the vanishing of the periodicity in molecular arrangement due to saturation and the nonlocal character of molecular reorientation (see e.g. the rightmost photos in Fig. 9b and c). Light propagation in the created waveguiding structure is shown in Fig. 10 with the significant difference in propagation characteristics for the light launched to the liquid crystalline waveguide channel with no voltage (Fig. 10b) and the voltage (Fig. 10c) applied to the periodic microchannel electrodes filled with EGaIn. As it can be noticed, for uniform orientation of molecules light is well confined in the core, whereas for electrically induced periodic alignment we can observe a quite strong scattering of light (few bright spots could be noticed outside core, where light is again scattered by walls of the electrode microchannel). Similarly, as for cylindrical-core waveguides, we were not able to observe “grating-like” spectra in LC: PDMS waveguides so far.

### Numerical simulations of spectral properties of LC-core waveguides with periodically aligned liquid crystals and qualitative analysis of the impact of the thermal fluctuations of the molecules

After unsuccessful attempts to observe “grating-like” spectra in both cylindrical and rectangular LC-core waveguides, we decided to perform some qualitative numerical analysis of such structures.

To present the main conclusion from our numerical analysis, we decided to choose a periodic structure characterized by the following nominal values of parameters: the length of the sample L = 20 mm, the period P = 20 µm (1000 periods), the same layer widths w_1_ = 10 µm and w_2_ = 10 µm, with corresponding values of the refractive index of n_1_ = 1.52 and n_2_ = 1.60 and filling factor FF = 0.5 (FF is defined as w_1_ / P).

**Figure 11:**
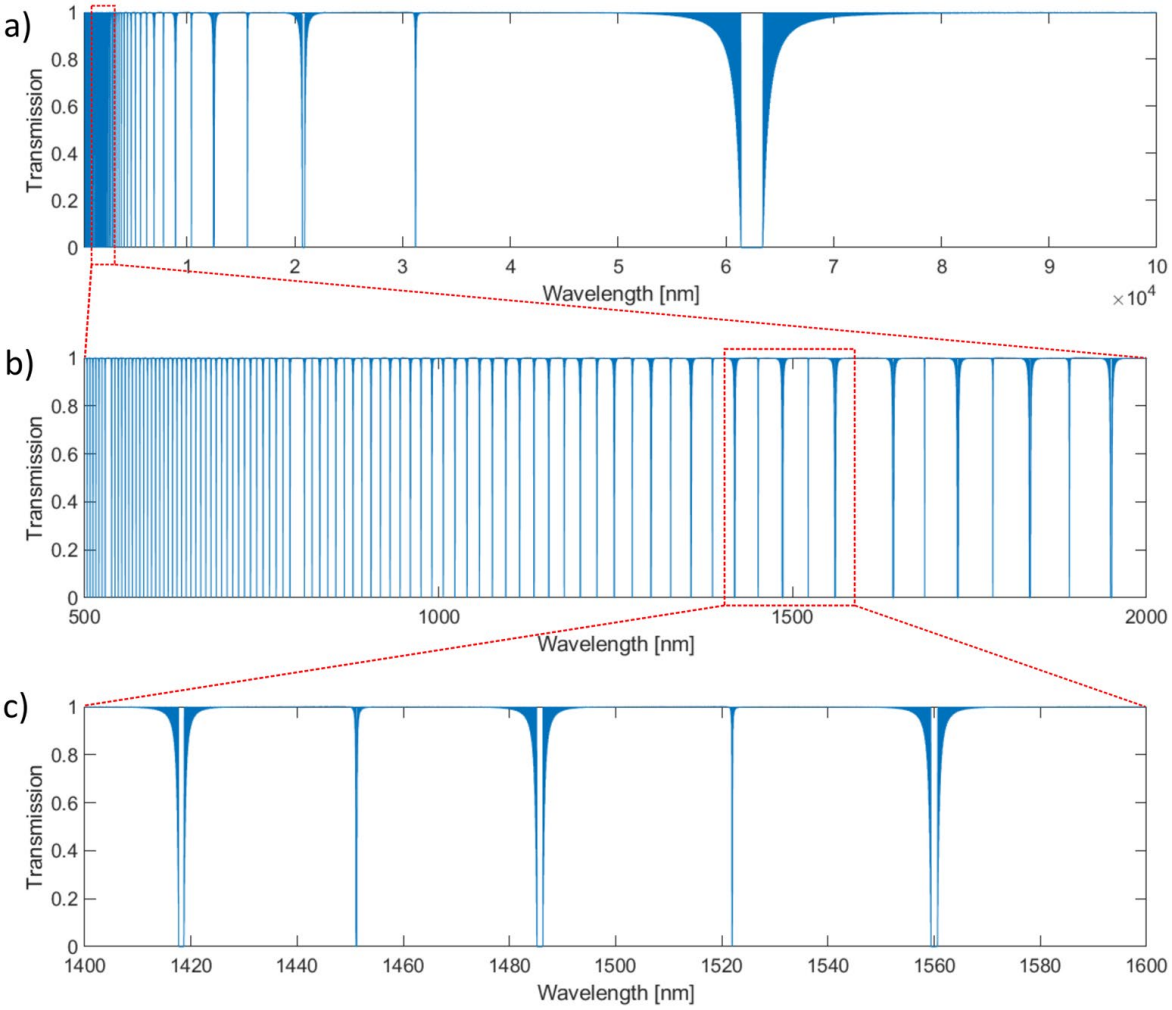
Simulated transmission spectra for “ideal” grating with period P = 20 µm, filling factor FF = 0.5, which means the same layer widths w_1_ = 10 µm and w_2_ = 10 µm, with corresponding values of refractive index of n_1_ = 1.52 and n_2_ = 1.60. All (**a**), (**b**) and (**c**) presents results calculated for the same sample, however (**b**) is “zoomed” to VIS–NIR range from 500 to 2000 nm, and (**c**) is “zoomed” to third telecom window from 1400 to 1600 nm (the spectral ranges used in measurements).

Figure 11 presents transmission spectra calculated for “ideal” structure – it can be noticed that the main band gap is located for the wavelength of about 60 μm, but in the visible and NIR regions, we should expect several dips in transmission spectra (in particular between 1400 and 1600 nm we should observe 3 dips with the spectral width of few nm).

**Figure 12:**
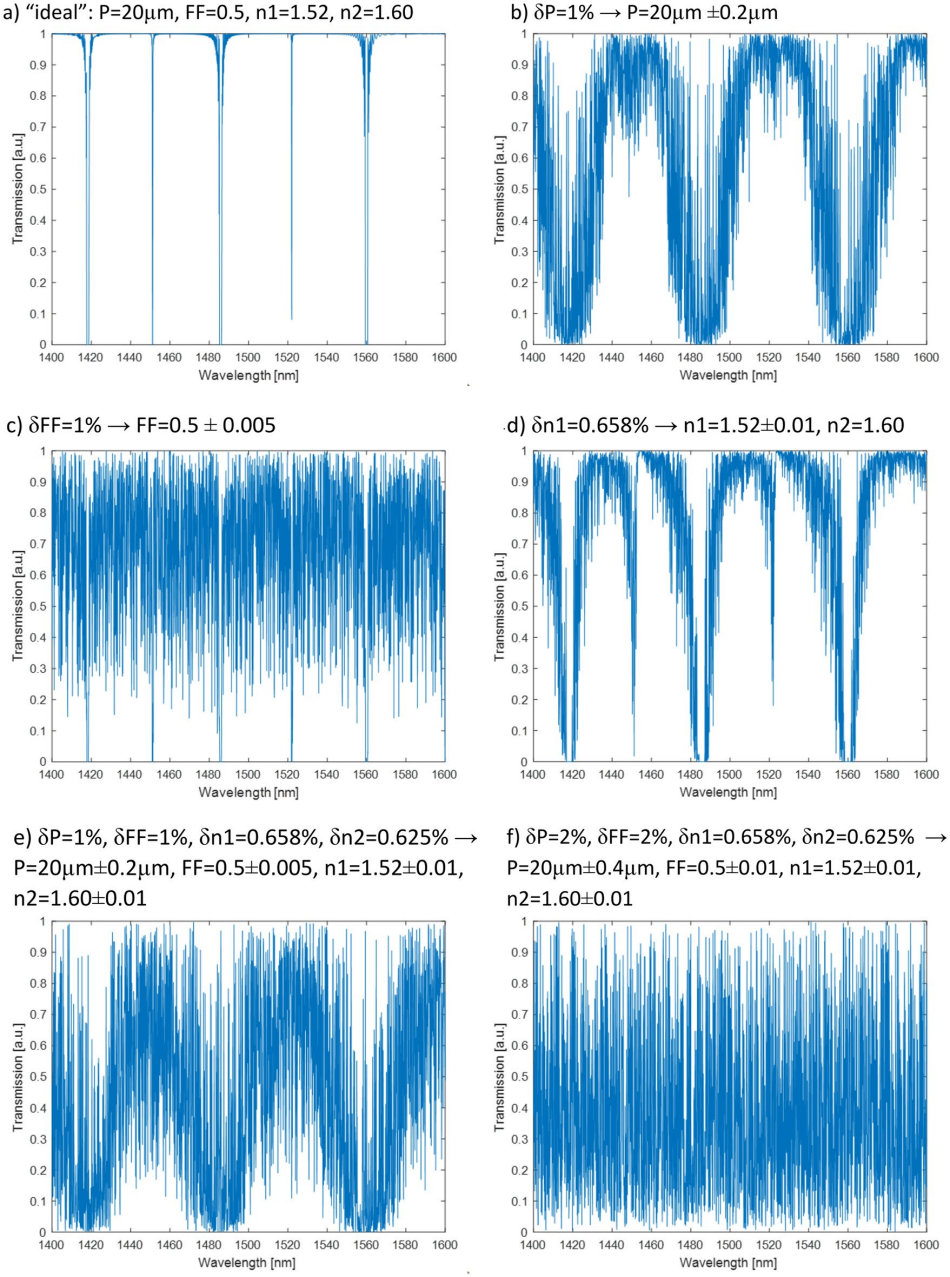
Exemplary results of numerical simulations comparing “ideal” spectra (**a**) with spectra affected by fluctuations (**δ**) in the sample period **P** (**b**), filling factor **FF** (**c**), refractive index—**n1** and **n2** (**d**), and all the mentioned parameters simultaneously (**e**) and (**f**).

In the next step, we decided to qualitatively simulate the impact of thermal fluctuations of liquid crystalline molecules. It was motivated by the observation of obtaining LC gratings with the polarizing microscope at exceptionally large magnifications. It was noticed that the obtained alignment is not perfectly stable, and some noticeable fluctuations of the LC molecules can be observed (unfortunately, the effect was too weak to be recorded by a camera). Such thermal fluctuations can lead to random changes in the effective refractive index, moreover, fluctuations at the borders between two differently aligned regions can result in fluctuations of the filling factor of grating or even its period. All those potential effects have been qualitatively investigated in our simulations. Exemplary results, based on the idealized structure described above, are presented in Fig. 12—all spectra were limited to the wavelength range 1400–1600 nm. Figure 12a presents spectra of the “ideal” undisturbed sample as a reference. Figure 12b shows the impact of 1% fluctuations of the grating period—the spectra are much noisier, but still some dips in the spectra could be expected. Figure 12c shows the impact of 1% fluctuations of the grating filling factor – the resulted spectra are even more noisy than in Fig. 12b, but some regular dips in spectra still could be noticed. The impact of refractive index fluctuations (in the range of ± 0.01) of only one type of the grating sections is presented in Fig. 12d – by comparing with Fig. 12b and c we can conclude that the impact of the refractive index fluctuations is slightly smaller than fluctuations of the period or filling factor. However, the resulted spectra are still much noisier than the “ideal” one. Figure 12e and f present the simulated impact of combined fluctuations, including the grating period, the grating filling factor, and both refractive indices. It could be noticed that in the case of strong (~ 2%) fluctuations of the filling factor (combined with resulting fluctuation of the period), the spectra become strongly noisy, and there is no trace of the original dips observed for the “ideal” structure.

### Spectral properties of the LC-core fiber microstructure with naturally induced periodicity

BPLC belongs to promising materials that may find applications in advanced photonic systems providing better transmission properties due to the existence of optical isotropy in BPLC. The latter results from an exotic arrangement of anisotropic molecules in the volume that creates self-assembling cubic nanostructures in the two phases BP I and BP II or occurs as an isotropic-like ‘fog phase’ BP III^62^. These indicate potential opportunities for BPLC to exploit in photonic systems enabling fast electro-optical modulation and switching, sensing physical and environmental parameters, as well tunable filter applications. Moreover, using BPLCs in fiber-optic systems can contribute to creating advanced and flexible systems operating in the visible spectrum that control the propagation properties^63–65^.

Basing on our experimental and theoretical experience, we were aware that successful realization of LC-core grating operating on higher-order band gaps could be a very challenging task (as higher order bandgaps are not only narrow, but as it appeared—they are very sensitive for any fluctuations). Our theoretical analysis indicated also, that first order band gap of the Bragg-type mirror/grating is less sensitive for fluctuations and other imperfectness. Consequently, we decided to create a fiber-optic microstructure in which light would be propagating in a “special” liquid crystal, which have natural tendency to create wide (first order) photonic band gaps. Generally, there are two types of LCs generating natural band gaps: chiral nematic (cholesteric) LCs that generate one-dimensional selective reflection and BPLCs that are known for selective reflection in all three-dimensions. 3D Bragg reflections in BPLCs are related to a cubic lattice structure with the size of the order of a few hundred nm and are characterized by sub-millisecond switching reaction times as well polarization insensitivity in a macroscopic scale for the wavelengths outside their resonance bands^66,67^. Notably, optical properties of BPLCs can be modified by external factors such as temperature, an external electric field, mechanical deformations, as well as nanoparticles doping^68–71^.

A LC mixture with induced blue phases (BPs) was prepared by adding chiral dopants to the host nematics (see “Methods”). The number of chiral dopants was chosen to achieve tunable selective reflections, covering the broadest range of Bragg wavelengths at different temperatures. The BPLC mixture was carefully introduced to a microcapillary with an inner diameter of 128 μm. Examination took place under a polarized microscope to provide a thin layer of LC filling that equaled around 40 μm.

**Figure 13:**
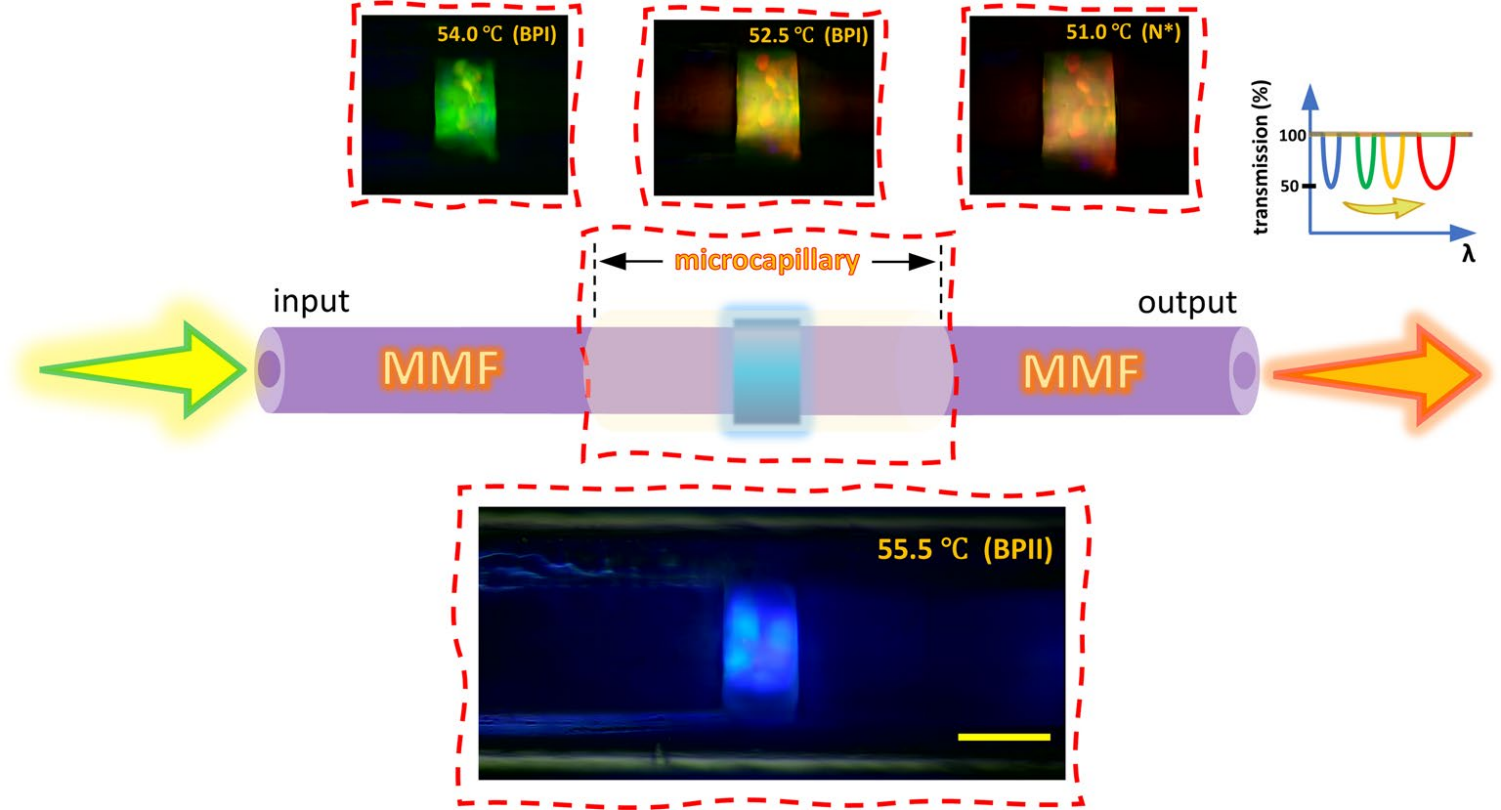
An idealized model of the working principle of the investigated fiber-optic microstructure with the BPLC microcavity. POM images present a longitudinal cross-section of the microstructure fiber with BPLC filling recorded in reflection. The color of the domains relates to Bragg reflection and depends on the LC phase at an appropriate temperature. A graph from the inset shows reduced transmission for the selected wavelengths corresponding to Bragg reflection that can be red shifted upon cooling the investigated BPLC microcavity in the fiber sample from BPII to N* phase. The yellow scale bar corresponds to 50 μm.

A fiber-optic microstructure with BPLC microcavity sensitive to temperature (Fig. 13) was developed by attaching two multimode fibers (MMFs) to the BPLC from both sides inside the microcapillary. When the BPLC filling in the fiber sample is cooled down from the isotropic phase, ISO (57.0 °C), passing through the subsequent BP phases to the chiral nematic phase, N* (51.0 °C and below), selective light reflection distinctly changes. This is caused by a change in periodicity of the chiral self-organization of LC molecules that manifests itself in switching color of the LC texture from blue to red, which relates to BPII and N* phases, respectively. Moreover, selective reflections for green and yellow light occurred in BPI at 54.0 °C and 52.5 °C, respectively, thus receiving complete control in a wide spectral range of photonic properties of the BPLC microcavity what is significant in our further investigations.

**Figure 14:**
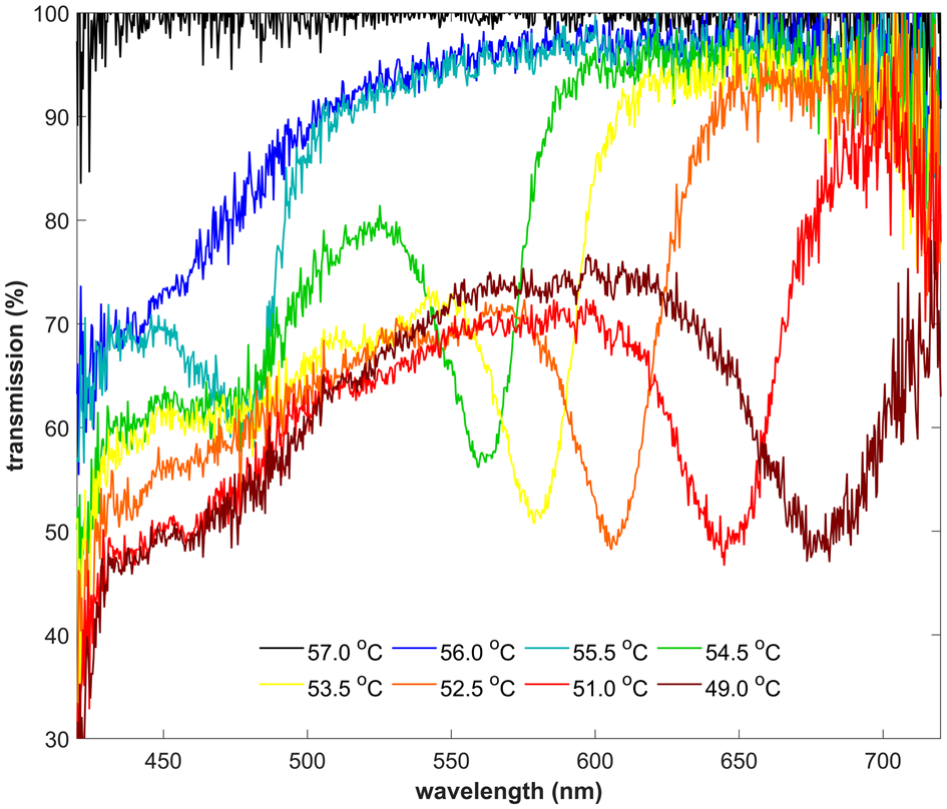
Spectra of the white light transmitted in the investigated microstructure fiber recorded upon cooling the BPLC microcavity from the isotropic phase (57.0 °C) to the chiral nematic phase (51.0 °C and below).

Interestingly, we found that it is possible to affect the transmission of the white light propagated in the fiber-optic microstructure by a thin layer of the aligned BP liquid crystals placed in the optical signal path. Figure 14 shows the spectral characteristics of the light guided in the investigated system. Upon cooling the sample from the isotropic phase to BPII, transmission becomes lower for the shorter wavelengths related to selective reflections of blue light. It is worth mentioning that during BP crystal nucleation near the ISO-BPII phase transition, the light is mainly scattered, what can be noticed as a spectrally broad decrease in transmission at 56.0 °C. Moreover, this effect also arises due to imperfection of BP crystal orientation in the bulk, revealing polycrystalline texture. At lower temperatures, the reduced transmission deriving from Bragg reflection shifts towards longer wavelengths, simultaneously increasing its spectral width from 24 nm (for BPII at 55.5 °C) up to 40 nm (for N* at 49.0 °C). Our investigations reveal broad tuning of photonic properties of the BPLC microcavity in the visible range from 475 to 678 nm, noting the wavelengths for the lowest transmission level in the spectra at the selected temperatures (LC phases). Unfortunately, the presented tuning of the photonic bandgap of the BPLC is not continuous throughout the entire aforementioned spectral range since a jump of the Bragg wavelength from 480 nm up to 560 nm occurs at the BPII-BPI phase transition. However, photonic bandgap tunability regarding BPI and N* phases is uninterrupted, for which the average thermal sensitivity of the examined BPLC microcavity corresponds to 22 nm/ °C.

Overall, orientation of LCs in the periodic waveguide structures is a crucial issue in achieving selective light reflections for the selected wavelengths. However, this is not so critical in the case of self-assembling chiral structures, where the imperfection of orientation of the BP domains can eventually increase the spectral width in the lower transmission signal corresponding to the Bragg reflection. It is also worth noting that our results unequivocally indicate that the detected Bragg reflections in the transmission spectra agree well with the color of the recorded BPLC textures at each LC phase, even though both observations were noted for two different directions. Therefore, we can say that the Bragg reflection band for aligned and not deformed BPLC domains is the same for perpendicular directions.

## Discussion

In this work, we demonstrated that reversible periodic photoalignment of the liquid crystal molecules is possible inside silica microcapillaries. We were able to control the period arbitrarily from about 500 μm down to 20 μm (in the contrast to previous works in the field when only few types of amplitude masks have been used for selective irradiation, typically one mask of period 600, 700 or 800 μm was used, i.e. Refs.^15,17,18,22^; only in Ref.^21^ three masks with period of 400, 500 and 600 μm have been used simultaneously). The obtained orientation was analyzed with polarized microscopy and we expected that propagation of a broadband light in such well-defined periodic structures should result in spectra typical for long-period fiber gratings or even fiber Bragg gratings. However, even that we created and measured a number of differently created samples (i.e., using different method of connections with SM fibers), we were not able to observe characteristic changes in transmission spectra that could be attributed to the created gratings. Our numerical model (although simplified—see “Methods”) can provide qualitative explanation of our unsuccessful experimental attempts for observation of the “grating-like” spectra. When we examined microcapillaries with periodic LC alignment under the polarizing microscope, at large magnifications we noticed that fluctuations of the borders between two different orientations of the LC molecules could have impact on the performance of the grating. Moreover, borders between two different orientations are not well defined, as resulted from our theoretical analysis, where “step-type” of the refractive index profile was assumed. In a real LC-core grating the border between two neighboring regions can be considered as “blurred”, as border between two different types of LC molecules orientation consists eighter continuous change of the molecules alignment, or some locally distributed topological defect—in both cases the border cannot be treated as “step-like” border between two regions with different refractive indices. As a result, it is much more difficult to obtain coherent reflection from such a “border,” and consequently it is much harder to obtain stable interference needed for creation of typical “dips” in the spectra.

**Figure 15:**
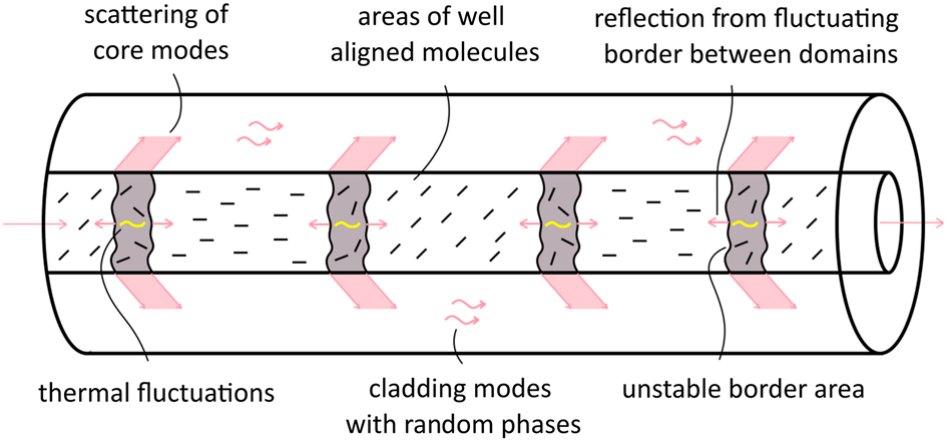
Schematical visualization of the main sources of the incoherent interference resulting in strongly random transmission spectra—“blurred” and fluctuating borders between two differently aligned LC core regions. As a result, not only core modes, but also cladding modes could be randomly induced and its interference with random phase will result in noisy spectra.

Moreover, there is also one factor that can disturb performance of the LC-core fiber grating, i.e., cladding modes and its random interference. At each interface between two differently oriented sections we can not only expect reflection of the light, by also some part of the light can be scattered and then propagate in the cladding as cladding modes, with a random phase, because scattering regions are “blurred”, and their borders fluctuate (Fig. 15). The more borders we have in the sample, the more randomly induced cladding mode could be induced, and consequently those cladding modes can randomly interfere at the sample output resulting with even more increased noise.

In this work, we have put a lot of effort in creating periodic LC structures within rectangular waveguides created by using PDMS. Although we were able to generate periodic changes of LC molecules alignment by using periodic microelectrodes, again, we were not able to measure transmission spectra typical to those observed in fiber gratings. The reasons could be probably the same as those described above for cylindrical LC-core waveguides.

Finally, we decided to evaluate waveguiding in LC with “naturally” defined periodicity obtained by using blue phase LCs. In this case, we were able to observe quite a broad dip in transmission spectra, corresponding to the first-order band gap spontaneously created by the BPLC (which periodicity is in order of few hundreds of nm). Moreover, by changing temperature, we were able to tune the band gap almost in the whole visible range. Observations of the BPLC with a microscope proved that molecules are not perfectly oriented, and some “domains” with slightly different shades could be noticed in Fig. 13. However, even with those “defects” we were able to obtain a quite noticable dip in the spectra. It was possible because the first-order band gap is much broader than higher-order band gaps, and moreover it is less sensitive to any imperfections in the structure—such conclusion was also confirmed by our numerical model when the stability of the first-order band gap was analyzed.

The results obtained for BPLC waveguide could provide a path for potential future optimization of tunable/reversible gratings created either with photoalignment or periodic electric field. It seems that it could be possible to obtain more stable grating-type spectra if the period of the gratings would be much smaller than we are currently able to obtain using our technologies. However, it seems to be reasonable to put some additional efforts in order to create effectively tunable (either by light or an electric field) LC-core waveguides with first-order Bragg gratings. In this work we used only nematic LCs and BPLCs, however interesting effects could be also expected if other types of LCs would be used, in particular ferroelectric LCs can be very useful for this purpose^72^.

## Data Availability

Data available based upon reasonable request from the corresponding author.
